# Older People’s Perceptions of what is Needed to Experience Dignity and Well-being at Residential Care Facilities

**DOI:** 10.1093/geroni/igab046.3507

**Published:** 2021-12-17

**Authors:** Charlotte Roos, Anna Swall, Lena Hammar, Anne-Marie Boström, Bernice Skytt

**Affiliations:** 1 Dalarna University, Falun, Dalarnas Lan, Sweden; 2 Mälardalen University / Dalarna University, Västerås, Vastmanlands Lan, Sweden; 3 Karolinska Institutet, Karolinska Institute, Stockholms Lan, Sweden; 4 University of Gävle, Gävle, Gavleborgs Lan, Sweden

## Abstract

Dignity and well-being are central values in care of older people living in residential care facilities. In addition, care of older people living in residential care facilities should be person-centred. Dignity and well-being can according to the person-centred practice framework be interpreted as person-centred outcomes. Despite this older people living in residential care facilities have described that they not fully experience dignity and well-being and improvements are needed. To improve care it is important to know what to target. The aim of this qualitative study was therefore to describe residents’ perceptions and experiences of what is needed to live with dignity and a sense of well-being. Interviews were carried out with older people living at residential care facilities (n=20). Inductive content analysis was used to analyse data and one overarching theme and three categories emerged. The result revealed the importance of, and that staff and the care environment supported, to manage daily life by oneself, to be shown respect and to belong to a social context. For older people to experience the person-centred outcomes dignity and well-being managers at residential care facilities need to develop and support the staff prerequisites related to knowledge, skills and attitudes and to improve the care environment. According to the person-centred practice framework, the staff prerequisites and the care environment must be taken into account to achieve the person-centred outcomes dignity and well-being.

